# Germline specification and axis determination in viviparous and oviparous pea aphids: conserved and divergent features

**DOI:** 10.1007/s00427-022-00690-7

**Published:** 2022-06-09

**Authors:** Gee-Way Lin, Chen-yo Chung, Charles E. Cook, Ming-Der Lin, Wen-Chih Lee, Chun-che Chang

**Affiliations:** 1 Laboratory for Genomics and Development, College of Bio-Resources and Agriculture, Department of Entomology, National Taiwan University (NTU), No. 1, Sec. 4, Roosevelt Rd., Taipei, 10617 Taiwan; 2 Department of Biochemistry and Molecular Cell Biology, School of Medicine, College of Medicine, Taipei Medical University, Taipei, Taiwan; 3 Research Center for Developmental Biology and Regenerative Medicine, NTU, Taipei, Taiwan; 4 Department of Molecular Biology and Human Genetics, Tzu Chi University, Hualien, Taiwan; 5 Research Center for Global SDGs Challenges, Office of Research and Development, Tzu Chi University, Hualien, Taiwan; 6 Institute of Biotechnology, College of Bio-Resources and Agriculture, NTU, Taipei, Taiwan; 7 Genome and Systems Biology Degree Program, NTU, Taipei, Taiwan; 8 International Graduate Program of Molecular Science and Technology, NTU, Taipei, Taiwan

**Keywords:** Parthenogenesis, Germ plasm, Vasa, *Hunchback*, Endosymbionts, Bacteriocytes

## Abstract

Aphids are hemimetabolous insects that undergo incomplete metamorphosis without pupation. The annual life cycle of most aphids includes both an asexual (viviparous) and a sexual (oviparous) phase. Sexual reproduction only occurs once per year and is followed by many generations of asexual reproduction, during which aphids propagate exponentially with telescopic development. Here, we discuss the potential links between viviparous embryogenesis and derived developmental features in the pea aphid *Acyrthosiphon pisum*, particularly focusing on germline specification and axis determination, both of which are key events of early development in insects. We also discuss potential evolutionary paths through which both viviparous and oviparous females might have come to utilize maternal germ plasm to drive germline specification. This developmental strategy, as defined by germline markers, has not been reported in other hemimetabolous insects. In viviparous females, furthermore, we discuss whether molecules that in other insects characterize germ plasm, like Vasa, also participate in posterior determination and how the anterior localization of the *hunchback* orthologue *Ap-hb* establishes the anterior-posterior axis. We propose that the linked chain of developing oocytes and embryos within each ovariole and the special morphology of early embryos might have driven the formation of evolutionary novelties in germline specification and axis determination in the viviparous aphids. Moreover, based upon the finding that the endosymbiont *Buchnera aphidicola* is closely associated with germ cells throughout embryogenesis, we propose presumptive roles for *B. aphidicola* in aphid development, discussing how it might regulate germline migration in both reproductive modes of pea aphids. In summary, we expect that this review will shed light on viviparous as well as oviparous development in aphids.

## Introduction

Aphids are small, soft-bodied, sap-sucking insects. Taxonomically, they constitute the superfamily Aphidoidea in the order Hemiptera. Developmentally, aphids are hemimetabolous: adults develop directly from nymphs without undergoing pupation or complete metamorphosis (Stern 2008). There are about 5000 named species (Blackman and Eastop 2006; Favret et al. 2016), of which at least 250 are agricultural and horticultural pests (Miller and Foottit 2017). With piercing mouthparts, aphids draw fluids from crops, resulting in plant dehydration. Additionally, aphids can serve as disease vectors, transmitting a diversity of plant viruses when moving from one plant to another while feeding (Ng and Perry 2004). Most of the extant aphid species reproduce asexually through parthenogenetic viviparity during at least part of their life cycle (Davis 2012), leading to rapid population expansions and concomitant economic damage.

**Fig. 1:**
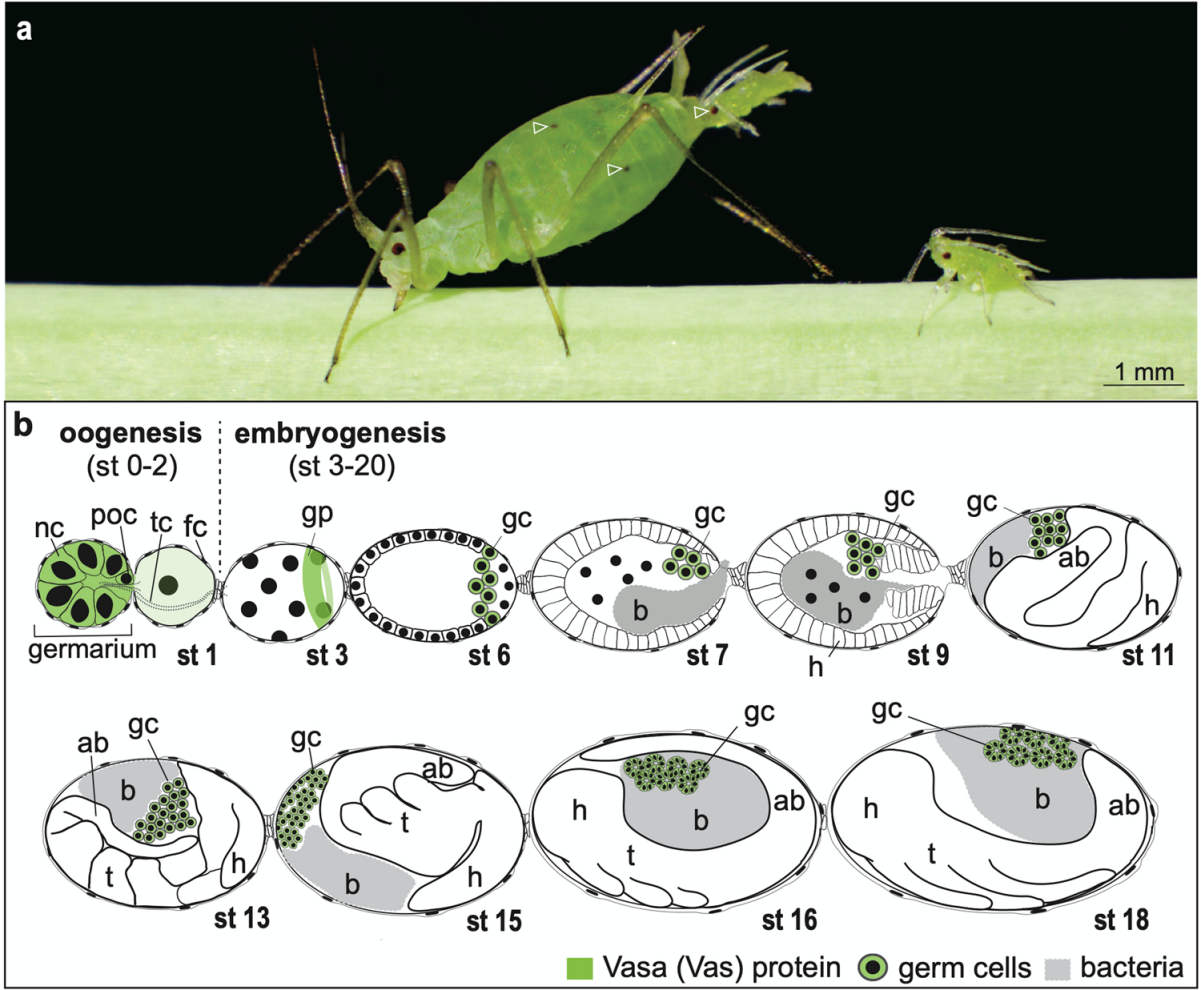
Embryonic development of the asexual (viviparous) pea aphid. **a** Image of a pea aphid (leftmost) that is giving birth via parthenogenetic and viviparous reproduction; a 1st-instar nymph is emerging from its gonopore. A newly born 1st-instar nymph (rightmost) is sucking plant sap. These two 1st-instar nymphs are born pregnant. Eyes of the hatching nymph and mature embryos within the abdomen of the mother are highlighted with hollow arrowheads. **b** Cartoon illustration of oogenesis and embryogenesis of the asexual pea aphid. All of the eggs accommodating oocytes and embryos are aligned within a telotrophic ovariole, where the germarium is situated at the anterior tip of the ovariole. Characteristics of the developmental stages follow Miura et al. (2003). Anterior of the eggs is to the left; dorsal is upper. Here, we do not display all of the stages but simply show representative morphologies that signify early, middle, and late embryogenesis. Stage 1: oocytes that are derived from prospective oocytes within the germaria. Stage 3: uncellularized syncytia containing multiple nuclei. Stage 6: cellularized blastoderm; the newly formed germ cells (marked in green) are located in the posterior region. Stage 7: late blastoderm being invaded by the endosymbiotic bacteria *Buchnera* (termed endosymbionts elsewhere) prior to gastrulation. Stage 9: invagination of the germ band—a developmental period after the blastoderm stage when onset of morphogenetic movements occurs. Stage 11: elongating embryos curled as an S-shaped germ band with distinctive head lobe morphology; by this stage, the endosymbionts are pushed to the egg anterior. Stage 13: elongating embryos with visible limb buds. In stage 14 (not shown), embryos become fully extended. Stage 15: embryos undergoing katatrepsis, through which embryos are flipped and the heads turn to the egg anterior. Stage 16: embryos after katatrepsis, where heads are settled in the egg anterior and the clustered germ cells are covered under the folded abdomen. Stage 18: embryos with a retracted abdomen after germ-band retraction in stage 17 (not shown). Vasa (Vas)-positive germ plasm can be identified in the posterior region of syncytia (see the stage 3 embryo). Before cellularization, nutrients and Vas are transported from germaria to syncytia through the trophic cords (Blackman 1978; Chang et al. 2006). Sizes of egg chambers, embryos, and cells at distinct stages are not shown according to scale. Abbreviations: ab, abdomen; b, bacterial endosymbionts; fc, follicle cells; gc, germ cells; gp, germ plasm; h, head; nc, nurse cells; poc, prospective oocyte; st, stage; tc, trophic cords; t, thorax. Color keys are under the illustration

The economic impact of aphids, as well as interest in the evolution of parthenogenesis, has attracted research interest for many decades. The pea aphid *Acyrthosiphon pisum* (Fig. 1a), as one of the first hemimetabolous species with a sequenced genome, has become a key species for studying comparative genomics across the insects. Among aphid species, *A. pisum* is a genomic model for exploring a range of molecular evolutionary traits for development as well as the metabolic basis of nutritional endosymbiosis (Mathers et al. 2017; The International Aphid Genomics Consortium 2010). Like many species of aphids, the pea aphid life cycle includes an asexual (viviparous) and a sexual (oviparous) phase. Thus, in one organism, it is possible to explore the unique and shared features of both developmental types. For example, there are striking spatiotemporal differences between viviparous and oviparous development. Viviparous embryogenesis is an order of magnitude faster than oviparous embryogenesis (approximately 10 days compared to 100 days; Miura et al. 2003; Shingleton et al. 2003). Viviparous eggs are also substantially smaller at the time of key patterning events in early development, with nearly a five-fold difference in egg length (Miura et al. 2003), and some key patterning differences have been described (Chung et al. 2018; Duncan et al. 2013). With the marked fecundity benefits of the asexual/viviparous mode, it is also worth noting that oviparous development is generally regarded as a seasonal feature that can be induced in the laboratory by controlling temperature and photoperiod (Ishikawa et al. 2011; Lin et al. 2014). Nevertheless, not every strain of a particular aphid species can be induced to lay eggs. Some pea aphid strains, for example, are not responsive to induction, skip oviparous development entirely, and are obligately parthenogenetic (Kanbe and Akimoto 2009). Indeed, we observe obligate parthenogenesis in the local strain of pea aphids that we handle in the laboratory (Lin and Chang 2016).

In this review, we describe what has been discovered about viviparous embryogenesis in this species. In particular, we focus on germline specification and axis determination, both of which are key developmental events during early embryogenesis. We seek to compare the differences between embryos that develop from viviparous oocytes and those that develop from oviparous oocytes. We then extend the comparison to several other established insect model organisms such as the fruit fly *Drosophila melanogaster* (Lynch et al. 2012), the cricket *Gryllus bimaculatus* (Donoughe and Extavour 2016), and the milkweed bug *Oncopeltus fasciatus* (Chipman 2017), all of which are exclusively sexual and oviparous.

## Germline specification in the pea aphid: an exception in the Hemimetabola

### Viviparous embryonic development in the pea aphid

Rapid expansion of pea aphid populations is enabled by asexual vivipary, usually during warmer months, in which unfertilized eggs undergo embryonic development within the eggs of telotrophic ovarioles, where oocytes and embryos are aligned in sequence as in an assembly line. The distinction between oocytes (stages 0–2) and embryos (stages 3–20) is determined by the appearance of the first nuclear division, which leads to the formation of the embryonic syncytium (stages 3, 4). After cellularization of the blastoderm (stages 5, 6), the maternal symbiotic bacterium *Buchnera aphidicola* invades the embryo through the egg posterior (stage 7). From gastrulation onward (stage 8), the posterior tip of the cellular blastoderm begins to invaginate into the interior part of the egg cavity. After that, part of the blastoderm becomes a thickened, segmented, and elongated embryo. The elongation of embryos occurs from stages 8 to 14 until just prior to the initiation of katatrepsis (embryo flip) (stage 15). Meanwhile, the remaining part of the blastoderm differentiates into the serosa, an extraembryonic membrane that enfolds the embryo. After katatrepsis, the elongated embryo retracts and the germaria become morphologically identifiable on the dorsal side. Germaria are located at the anterior end of ovarioles and contain undifferentiated cells that will form nurse cells and oocytes: in the viviparous embryos, their presence indicates that the nurse cells and the oocytes are about to differentiate. We have observed that the mature embryos (daughters; stages 16–20), in turn, already bear blastoderm stage embryos (granddaughters) (Chang et al. 2007). Hence, the 1st instar nymphs (Fig. 1a) are born pregnant. This “Russian doll” type of reproduction contributes to an exponential increase of offspring among viviparous aphid species (Stern 2008). Characteristics of developmental stages are outlined and illustrated in Fig. 1b (Miura et al. 2003).

### Pea aphids possess a preformed germ plasm expressing Vas

**Fig. 2:**
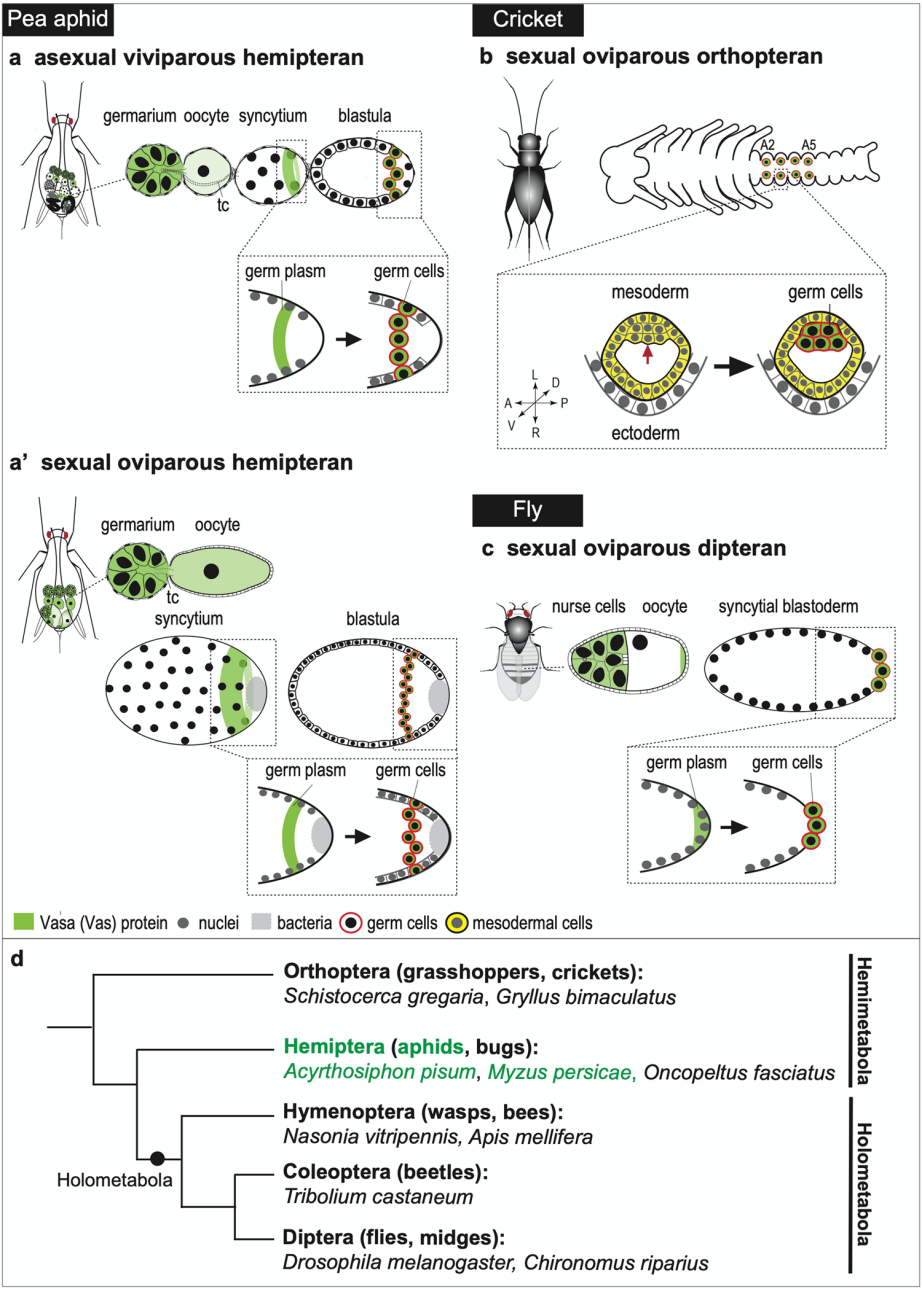
Comparison of germline specification in pea aphids, flies, and crickets. **a** Asexual (viviparous) pea aphid, hemipteran. **a'** Sexual (oviparous) pea aphid. In both viviparous and oviparous embryos, Vas-positive germ plasm in the posterior region of the eggs can be identified and Vas is later incorporated into the newly formed germ cells. The difference between viviparous and oviparous embryos is the timing of the appearance of germ plasm: in viviparous embryos, germ plasm can be clearly visualized from the first round of nuclear division onward whereas in oviparous embryos germ plasm cannot be detected until the fifth nuclear division is completed. **b** Cricket, orthopteran. In *Gryllus*, a Vas-positive germ plasm is not detected in any subcellular regions of the oocytes or syncytia. Instead, Vas-positive signals are detected in abdominal segments 2 to 5 in fully segmented embryos (Ewen-Campen et al. 2013a). The inset (abdominal segment 3) shows that specification of germ cells depends on signal induction (arrow). Anterior (A)–posterior (P), dorsal (D)–ventral (V), and left (L)–right (R) axes are annotated beside the magnified illustrations. **c** Fly, dipteran. In *Drosophila*, the Vas-positive germ plasm is established in the posterior pole of the oocytes during mid-oogenesis. After fertilization, Vas protein in the germ plasm is inherited by the first cellularized cells, namely, the germ (pole) cells, residing in the egg posterior. The relationships between germ plasm and newly formed germ cells in aphids and flies are magnified in the insets. Color keys and symbols are highlighted under panels **a** to **c**. **d** A phylogeny showing insect species mentioned in this review, based upon Misof et al. (2014)

In the asexual pea aphid, each embryo initially derives from prospective oocytes located at the posterior end of the germarium (also termed tropharium in the telotrophic-type ovariole; Büning 1985). The maturing oocytes maintain a cytoplasmic connection to, and receive nutrients from, the nurse cells of the germarium throughout oogenesis and early embryogenesis (Fig. 1b). Further investigation of how germ cells are specified will clarify how viviparous embryos originate. We studied germ-cell specification by staining embryos using an antibody against Vasa (Vas), an ATP-dependent RNA helicase that is a highly conserved germline marker in animals (Raz 2000). Immunostaining data shows that morphologically identifiable germ cells in the fully-segmented embryos are specifically labeled, indicating that the pea aphid also uses Vas as a conserved germline component (Chang et al. 2006). Through careful inspection of Vas protein localization throughout earlier developmental stages, prior to the germ cells residing in the gonads, we identified a presumptive germ plasm—a cytoplasm containing germline determinants—in the posterior region of the syncytial blastoderm (Chang et al. 2006). Together, these results strongly suggest that germline specification in the viviparous pea aphid is driven by a preformed germ plasm (Figs. 1b, 2a).

This germ plasm–driven mode, however, is not unique to viviparous reproduction in pea aphids. Vas-positive germ plasm can also be detected in the posterior region of syncytia with 32 dividing nuclei in pea aphids developing from the sexual phase of their life cycle (Lin et al. 2014). In comparison with the viviparous germ plasm, emergence of the oviparous germ plasm requires four additional rounds of nuclear division. Despite these additional nuclear divisions, in both viviparous and oviparous embryos the germ plasm is assembled during early embryogenesis while the syncytium is forming (Fig. 2a, a’) (Chang et al. 2006; Lin et al. 2014). Whether Vas that is restricted to the newly assembled germ plasm is purely maternal or partially zygotic remains uncertain. However, two pieces of evidence suggest that it is maternal Vas in the germ plasm of both viviparous and oviparous embryos: (1) expression of Vas can be continuously detected in mature oocytes as well as in newly laid eggs using both in vitro (Western blots) and in vivo (immunostaining) assays, and (2) a significant increase of Vas is not identified until the invagination of the blastoderm, suggesting that zygotic Vas is actively synthesized only from gastrulation onward (Chang et al. 2006; Lin et al. 2014).

### Comparison of germline specification between pea aphids and other hemimetabolans

As determined by the presence of Vas and other germ plasm markers, the germ plasm–driven mode of germline specification in the viviparous and oviparous pea aphids is so far unique within the hemimetabolous insects (Fig. 2d). Among the established models of hemimetabolans, maternally inherited germ plasm is not detectable and germ cells are observed to be segregated from somatic cells through signal induction (Ewen-Campen et al. 2013a; Extavour and Akam 2003). For example, in the cricket *Gryllus bimaculatus*, germline segregation is initially detected in the abdomen following segmentation, with germline clusters appearing in abdominal segments 2 to 5 (Fig. 2b). A maternal germ plasm is not identified per se but mesodermal disruption by *twist* RNAi can eliminate the formation of germ cells, suggesting that germline specification in crickets depends on signals released from mesodermal cells (Ewen-Campen et al. 2013a; Mito et al. 2005). Similarly, in the grasshopper *Schistocerca gregaria*, an orthopteran like *G. bimaculatus* (Fig. 2d), germline segregation is not detected in the abdomen until it is segmented (Chang et al. 2002). In the milkweed bug *Oncopeltus fasciatus*, a hemipteran like aphids (Fig. 2d), germ cells initially segregated during early gastrulation can be labeled by the antisense riboprobe of Vas using whole mount in situ hybridization (WISH). However, WISH results for Vas and 18 other conserved germline genes do not mark a maternal germ plasm (Ewen-Campen et al. 2013b). Taken together, it suggests that the signal induction mode, which is a more ancestral mechanism than the germ plasm–driven mode (Extavour and Akam 2003), prevails in the hemimetabolous insects. Therefore, the germ plasm–driven mechanism identified in aphids is likely to be a derived feature.

### Comparison of germline specification between pea aphids and *Drosophila*

In some holometabolous insects such as *Drosophila*, germ plasm is assembled during oogenesis (Fig. 2c, d) (Lynch et al. 2011). In the pea aphid, a hemimetabolan, assembly of the germ plasm is observed to take place later during embryogenesis (Chang et al. 2006; Lin et al. 2014). However, both *Drosophila* and the pea aphid share a common feature: establishment of a posteriorly localized germ plasm using maternal Vas proteins. The germ plasm forms prior to cellularization, which is termed “preformed.” This is observed regardless of whether the germ plasm is established within an oocyte or an embryo. Nevertheless, experimental results based upon ectopic expression of the pea aphid *vas *gene (*Ap*-*vas1*) in *Drosophila* suggest that formation of the maternal germ plasm in these two species arises through different molecular networks (Wang et al. 2015).

The Vas protein is a conserved germ plasm component in both *Drosophila* and the pea aphid, but the mechanisms by which Vas is localized are clearly different. In *Drosophila*, both *oskar* (*osk*) mRNA and Osk protein are molecular anchors that localize other germ plasm components, including Vas. Localization of *osk*/Osk to the posterior pole of oocytes initiates assembly of the germ plasm (Ephrussi et al. 1991; Ephrussi and Lehmann 1992; Kim-Ha et al. 1991). The pea aphid genome, however, does not have an orthologue of *Drosophila osk* (Shigenobu et al. 2010; The International Aphid Genomics Consortium 2010). This suggests that the assembly of germ plasm in the pea aphid, unlike *Drosophila*, is *osk*-independent. Ectopic expression of the pea aphid Vas (*Ap*-Vas1) in *Drosophila* oocytes, unlike the endogenous Vas (*Dm*-Vas), fails to localize to the germ plasm, confirming the *osk*-independence of *Ap*-Vas1 (Wang et al. 2015). Our further analysis shows that the amino acid glutamine (Gln) 527 in the conserved HELICc domain of Vas is critical to its Osk-dependent localization to the germ plasm. For example, the presence of Gln527 in *Dm*-Vas and the grasshopper *Schistocerca* Vas (*Sg*-Vas) facilitates successful localization of both Vas proteins to the germ plasm in *Drosophila*. By contrast, *Ap*-Vas1, *Gryllus* Vas, and *Oncopeltus* Vas, all of which lack Gln527, do not localize in the *Drosophila* germ plasm (Wang et al. 2015). In *Schistocerca*, neither an *osk* homolog nor a maternal germ plasm can be identified (Chang et al. 2002), but *Sg*-Vas with Gln527 does localize to the Osk-containing germ plasm in *Drosophila*, implying that this change is unrelated to the evolutionary acquisition of Osk. Whether there is a “non-Osk” molecule to anchor *Ap*-Vas1 to the germ plasm or *Ap*-Vas1 itself acts as a molecular anchor to assemble the germ plasm in pea aphids requires further investigation.

### Germline migration in the pea aphid: two special features

**Fig. 3:**
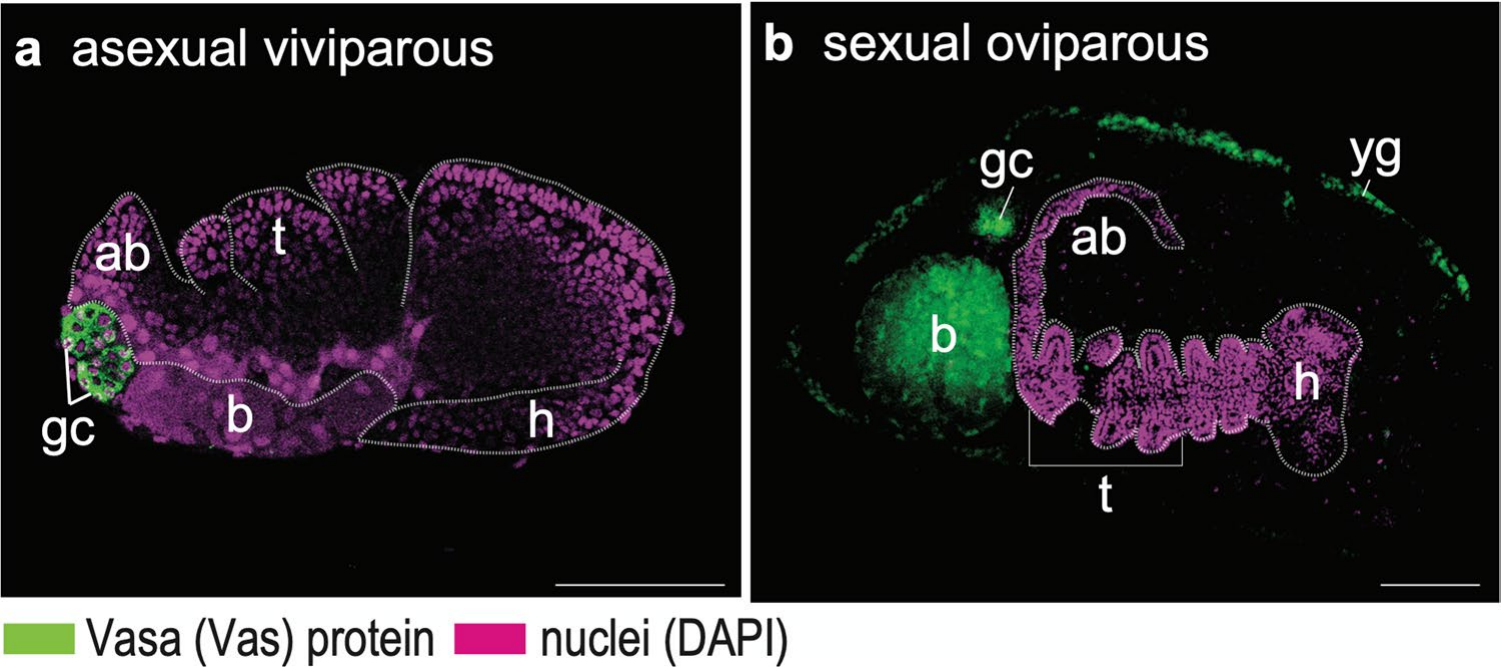
Locations of migrating germ cells during mid-embryogenesis of the pea aphid. **a** Viviparous embryos undergoing katatrepsis (stage 15). **b** Oviparous embryos at 14–16 days after egg laying, where segmentation is almost complete. Egg anterior is to the left; embryonic anterior is the head. An antibody against *Ap*-Vas1 was used to label the embryonic germ cells. In the viviparous embryos, germ cells located to the egg anterior are specifically stained. In the oviparous embryos, morphologically identifiable germ cells beside the extending abdomen are labeled. The Vas-positive germ cells within both viviparous and oviparous eggs, according to the staining results, are located external to the embryos. Signals observed in the endosymbionts and yolk granules are endogenous autofluorescence, which are *Ap*-Vas1 negative. Embryo shapes are highlighted by dashed lines. Color keys are under the panels. Abbreviations: ab, abdomen; b, bacterial endosymbionts; gc, germ cells; h, head; t, thorax; yg, yolk granules. Scale bars: 100 μm

**Fig. 4:**
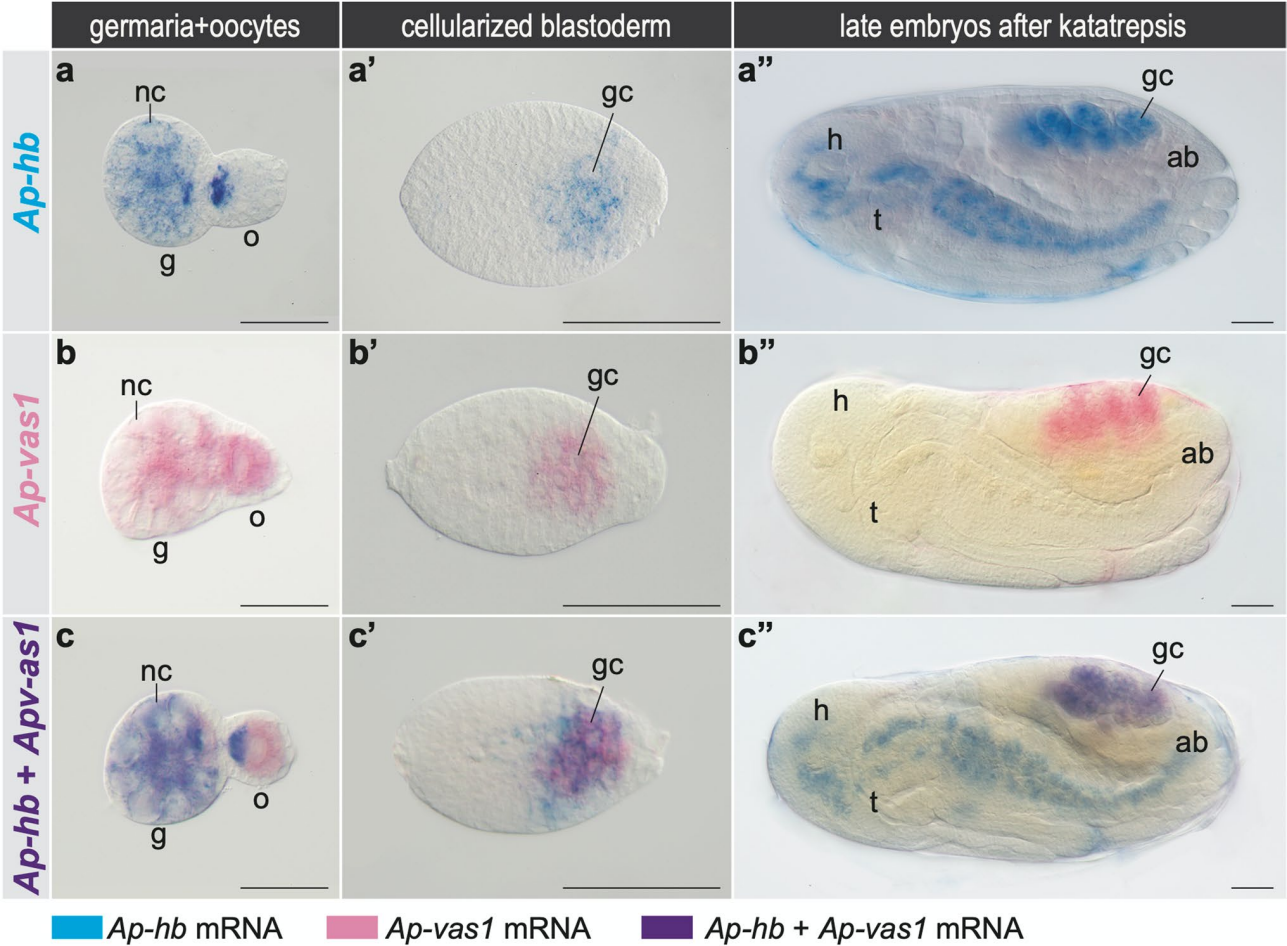
Germline expression of *Ap-hb* and *Ap-vas1* mRNAs during oogenesis and embryogenesis in asexual pea aphids. Gene expression images are from WISH. Selected stages shown here include germaria and stage-1 oocytes (**a**–**c**), stage-6 blastoderm (**a'**–**c'**), and stage-18 late embryos (**a"**–**c"**). Anterior is left; dorsal is upper for late embryos undergoing germ-band retraction. Color keys are under the panels. **a**–**a"** *Ap-hb* expression. **b**–**b"** *Ap-vas1* expression. **c**–**c"** Colocalization of *Ap-hb* and *Ap-vas1*. Both transcripts are co-localized in the germline lineage, which includes germaria (**c**) and germ cells in blastoderm (**c'**) and late embryos (**c"**). Although both *Ap-hb* and *Ap-vas1* mRNAs are expressed in oocytes, anterior localization only occurs for *Ap-hb* (**a**, **c**). Apart from germline expression, *Ap-hb* is transcribed in neuroblasts along the AP axis in late embryos (**a"**, **c"**). Abbreviations: ab, abdomen; g, germarium; gc, germ cells; h, head; nc, nurse cells; o, oocyte; t, thorax. Scale bar: 50 μm

In all insects studied to date, germ cells are specified first, then migrate (Santos and Lehmann 2004). This also applies to the pea aphid: germ cells follow a programmed migration to reach the gonadal mesoderm to form a functional gonad (Chang et al. 2007). In both asexual and sexual pea aphids, germline migration shares two characteristics that may distinguish aphids from other insects, including holometabolans. First, germ cells remain external to the embryos until katatrepsis initiates (Fig. 3). The newly formed germ cells remain outside the blastoderm after they are specified in the egg posterior (Figs. 2a, a'; 4a’–c’). Then, while the germ band is elongating, germ cells are pushed toward the egg anterior by the extending abdomen. During the embryonic inversion events of katatrepsis, the germ cells—along with the abdomen—return to the egg posterior, where the germ cells finally start to invade the interior of the embryo. After the embryonic head settles in the egg anterior, namely, the completion of katatrepsis, germ cells reside bilaterally in the dorsal region of the abdomen (Chang et al. 2009; Chang et al. 2007; Lin et al. 2014). At the same time, formation of germaria is underway (Fig. 1b, stage 16). Second, migration of the germ cells is associated with the invading endosymbionts from gastrulation onward. We have also identified the germline-endosymbiont association in the green peach aphid *Myzus persicae* (Chung et al. 2018), suggesting that this special phenomenon is conserved among aphid species.

In *Drosophila*, the primordial germ cells, or pole cells, are specified at the posterior pole of the embryos prior to blastoderm formation (Hay et al. 1988; Lasko and Ashburner 1988). When gastrulation begins, the specified germ cells are soon brought inside the embryonic cavity by their association with the invaginating posterior midgut primordium (Santos and Lehmann 2004). In contrast to *Drosophila*, pea aphids preserve migration of germ cells outside the embryos much longer. Results from immunostaining and WISH (Fig. 3a; Chang et al. 2007) show that germ cells remain external and are located adjacent to the extending abdomen of the viviparous embryos until the beginning of katatrepsis at stage 15 (Fig. 3a), long after the initiation of gastrulation at stage 8 of development (Miura et al. 2003). Similar locations of germ cells are also identified nearby the abdomen until early katatrepsis in the oviparous embryos (Fig. 3b; Lin et al. 2014). We suggest that these differences between aphids and flies may relate to heterochronic consequences of their modes of segmentation. In *Drosophila*, specification of all segments (parasegments) takes place within a short time window during cellularization of the blastoderm (Akam 1987). Because the body plan is established early, specification of the gonadal mesoderm is also determined comparatively early during late gastrulation (Boyle and DiNardo 1995; Van Doren et al. 1998). Accordingly, germline migration into the somatic gonads is known to proceed from gastrulation onward. In the pea aphid, by contrast, segmentation is not complete until the end of abdominal extension (Miura et al. 2003). Hence, the germ cells may not have a complete embryo in which to take up residence until the gonadal precursors are well-formed within the abdomen. For this reason, germ cells may be forced to remain external to the embryo in aphids until segmentation is complete just prior to katatrepsis.

At present, we can only conclude that presence of the morphologically identifiable germaria dorsal to the embryo indicates the completion of coalescence between germ cells and somatic gonads (Will 1888; Hagan 1951; Chang et al. 2007). When and where the germ cells and gonadal mesoderm first encounter each other remains uncertain, in part due to outstanding uncertainty on when bone fide germarium formation is complete. Migrating germ cells may form germ-cell clusters first and then coalesce with the gonadal mesoderm, or vice versa. Finding a specific marker to label the gonadal mesoderm, together with tracing the migrating germ cells expressing *Ap*-Vas1, will be helpful to understanding the formation of functional gonads in both asexual and sexual pea aphids.

With regard to the germline-endosymbiont association, we propose two potential roles of *Buchnera* in regulating nutrition and migration for germ cells. First, *Buchnera* may provide nutrients to the migrating germ cells, sustaining their development (Douglas 1998; Feng et al. 2019). Second, *Buchnera* may act as mediators to transmit signals released from somatic gonads, which are differentiated from the somatic mesoderm, to guide the migration of germ cells. These two roles are not mutually exclusive and *Buchnera* may serve both functions. If this is the case, *Buchnera* may spend relatively more energy on nutrient provision during early gastrulation when gonadal mesoderm has not yet formed. Later, even while transmitting guiding signals from the somatic gonads, *Buchnera* could still continue to provide required nutrients to the migrating germ cells.

Bacteriocytes, which are the specialized host cells that host *Buchnera*, may also participate in the transmission of signals for guiding germ-cell migration. In fact, it is bacteriocytes, rather than *Buchnera*, that directly contact the migrating germ cells in the pea aphid (Braendle et al. 2003). Guiding signals sent out from the somatic gonads may penetrate through the bacteriome—an organ-like collection of bacteriocytes—or, alternatively, the bacteriome itself might produce intermediate signals to guide the germ cells to migrate on the right path. In *Drosophila*, we have not found descriptions of the interaction between germ cells and bacteriocyte-like cells (Sacchi et al. 2010). However, the relationship between germline development and obligate endosymbiosis has been studied for the endosymbiont *Blochmannia* and their ant hosts of the hyperdiverse ant tribe Camponotini. According to Rafiqi et al. (2020), *Blochmannia* regulates the expression of germline genes like Vas through the *Hox* genes *Abdominal A* (*abdA*) and *Ultrabithorax* (*Ubx*). RNAi knockdown of *abdA* and *Ubx* results in elimination or malposition of bacteriocytes and germ cells. This suggests that the *abdA*/*Ubx*-positive bacteriocytes are involved in regulating germline development in the ants. In the seed bug *Nysius plebeius* and the pea aphid *A. pisum*, expression of *abdA* and *Ubx* can also be identified in bacteriocytes (Braendle et al. 2003; Matsuura et al. 2015), implying that the *abdA*/*Ubx*-dependent maintenance of bacteriocytes, as in ants, may be conserved and required for sustaining germline development.

## Axis determination in the pea aphid: conserved and divergent aspects

### Asymmetric localization: a strategy for specifying the AP axis in insects

**Fig. 5:**
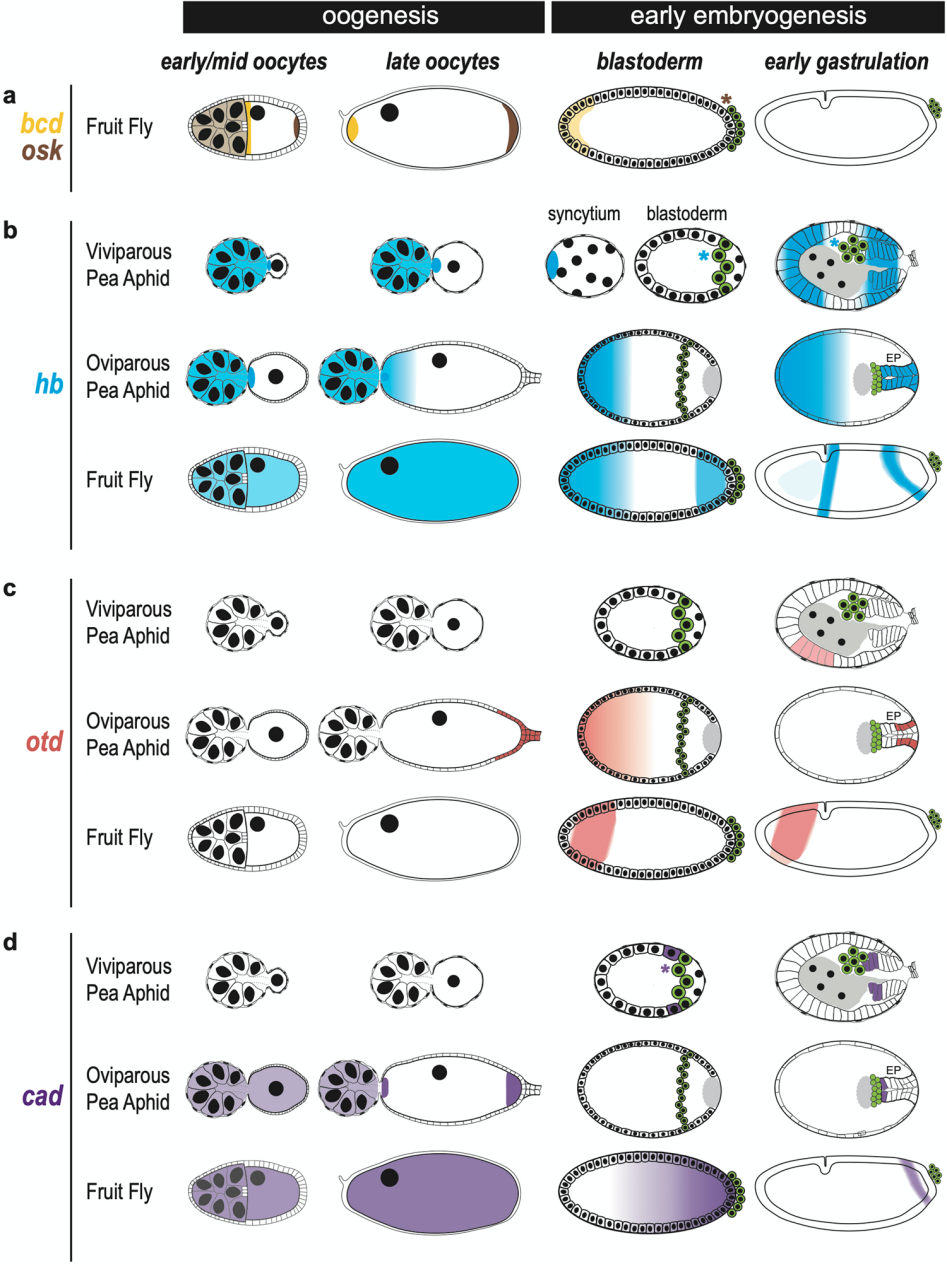
Comparison of gene expression along the anterior-posterior axis in asexual pea aphids, sexual pea aphids, and fruit flies during oogenesis and early embryogenesis. Target genes include *bicoid* (*bcd*), *oskar* (*osk*), *hunchback* (*hb*), *orthodenticle* (*otd*), and *caudal* (*cad*). Expression patterns show the distribution of gene transcripts. Vas-positive germ cells, as shown in Figs. 1 and 2, are marked in green. Abbreviations for each gene are in the same color used to show that gene’s expression. Schematics are mid-sagittal, with the anterior of eggs to the left and dorsal up for species and stages at which this can be determined (uncertain for oviparous pea aphids). In asexual aphids, early oocytes are emerging from the germarium; late oocytes are the eggs that will begin embryogenesis once nuclear division begins. **a** *bcd* and *osk*. Expression of both *bcd* and *osk* is detected in *Drosophila* but not in aphids. Maternal *bcd* and *osk* are synthesized by the nurse cells. Asymmetric localization of *bcd* (anterior) and *osk* (posterior) starts from mid-oogenesis onward. In early blastoderm, anterior localization of *bcd* becomes weak whereas *osk* is restricted within the pole cells. The brown asterisk adjacent to the germ cells indicates germline expression of *osk*. In embryos undergoing gastrulation, localization of both *bcd* and *osk* transcripts is not detected (BDGP 2021; Ephrussi and Lehmann 1992). **b** *hb*. In aphids and flies, maternal *hb* mRNA is synthesized by the nurse cells but anterior localization of *hb* can only be identified in the aphid oocytes. In the oocytes of flies, maternal *hb* is initially uniformly distributed. During aphid embryogenesis, localization of *hb* to the anterior pole can be identified only in the viviparous syncytium but it is absent in the blastoderm. In oviparous aphids and fly embryos, *hb* is strongly expressed in the anterior. During early gastrulation, expression of *hb* can be detected in both anterior and posterior regions of viviparous aphids and fly embryos. In oviparous development, strong expression of *hb* can be identified in the egg anterior but *hb* is uniformly expressed in the embryonic primordia. To emphasize germline expression of *hb*, blue asterisks are shown beside the Vas-positive germ cells in green in the viviparous embryos (Chung et al. 2018; Duncan et al. 2013; Huang et al. 2010; Margolis et al. 1994; Tautz et al. 1987). **c** *otd*. In germaria and oocytes of aphids and flies, *otd* expression is not detected except in the posterior follicle cells of oviparous oocytes of sexual pea aphids. During embryogenesis, anterior expression of *otd* in the egg anterior can be detected in blastoderm of oviparous aphids and flies; however, a similar pattern cannot be observed in blastoderm of viviparous aphids. During early gastrulation, anterior expression of *otd* can be identified in aphids and flies (Duncan et al. 2013; Gao et al. 1996; Huang et al. 2010). **d** *cad*. Maternal *cad* is not detected in germaria and oocytes of viviparous aphids but *cad* expression in germaria and oocytes can be observed in oviparous aphids and flies. In late oocytes, anterior and posterior localizations of *cad* in oviparous aphids are detected but universal expression of *cad* is observed in flies. During embryogenesis, posterior expression of *cad* can be identified in both aphids and flies. A notable difference between aphids and flies is that *cad* expression is not detected until gastrulation in the oviparous aphids but flies, like asexual aphids, display posterior expression of *cad* earlier during cellularization of the blastoderm. In the cellular blastoderm, expression of *cad* can even be identified in the newly formed germ cells (purple asterisk) (Chang et al. 2013; Duncan et al. 2013; Mlodzik and Gehring 1987). Abbreviation: EP, embryonic primordium

In some insects, the embryonic anlage extends along the entire egg and all segments are determined almost simultaneously during early embryogenesis (St Johnston and Nüsslein-Volhard 1992). These “long-germ” insects usually establish the anterior-posterior (AP) axis via asymmetric localization of maternal determinants to the opposite poles of the syncytial embryos (Nüsslein -Volhard and Roth 1989; St Johnston and Nüsslein-Volhard 1992). In *Drosophila*, for example, anterior localization of *bicoid* (*bcd*) mRNA and posterior localization of *nanos* (*nos*) mRNA during mid-oogenesis will determine the anterior and posterior poles, respectively, of the resulting embryo (Fig. 5a) (Kugler and Lasko 2009; McGregor 2005). During mid-oogenesis, the Osk protein that is translated from the posteriorly localized *osk* mRNA begins recruiting germline components such as *nos* mRNA into the germ plasm. This posterior localization of Nos thus provides another molecular marker, in addition to *bcd* and *osk*, for marking AP polarity of the oocytes (Lasko 2012).

Although the strategy of asymmetric localization is also employed by other long-germ insects, molecular networks for specifying the AP axis appear diverse, particularly among species without a *bcd* orthologue. Even more strikingly, there is evidence for species using fundamentally different sets of molecules to disrupt egg symmetry and form the AP axis. For example, in the mosquito-like midge *Chironomus riparius*, which lacks a *bcd* orthologue, mRNA of the cysteine-clamp gene *panish* is anteriorly localized in the syncytial blastoderm (Klomp et al. 2015). RNAi of *panish* results in abdominal development at both embryonic poles, but ectopic expression of *panish* at the posterior pole does not generate a second head in the posterior. Additionally, disruption of the posteriorly localized Nos, unlike *Drosophila*, does not affect axial patterning. These results suggest that *Chironomus* utilizes a distinct pathway to generate AP polarity in which the *panish* protein acts as a repressor for posterior genes in the anterior and the Nos protein realizes other functions beyond its known roles for posterior patterning in *Drosophila* (Klomp et al. 2015). In the wasp *Nasonia vitripennis*, another long-germ insect lacking *bcd*, *orthodenticle* (*Nv*-*otd1*) mRNA is first localized to the oocyte posterior during early oogenesis and, then, from mid-oogenesis onward, *Nv*-*otd1* mRNA is restricted to the oocyte anterior (Lynch et al. 2006). In addition to the posterior localization of *Nv*-*otd1*, transcripts of *Nv*-*Nos*, *Nv-cad*, and *Nv-osk* are also localized to the oocyte posterior, with all contributing to form the posterior region in *Nasonia* (Lynch and Desplan 2010; Lynch et al. 2011; Olesnicky et al. 2006). Together, these observations suggest that different long-germ insects make use of distinct gene regulatory connections among conserved patterning factors for directing AP patterning.

Unlike long-germ insects, the embryonic anlage of the short/intermediate germ insects—in which abdominal segments are progressively added after gastrulation—only occupies part of the egg. In the beetle *Tribolium castaneum*, a short/intermediate insect model, maternal *Tc-germ cell-less*, is known to direct the anterior localization of maternal *Tc-axin* (Ansari et al. 2018). After *Tc-axin* mRNA is maternally inherited by the fertilized egg, it promotes the zygotic expression of anterior genes such as *Tc-homeobrain* and *Tc-zen1*. Formation of the posterior region relies on *Tc-cad* and Wnt, both of which are operating zygotically in the growth zone. According to Ansari et al. (2018), although the initial asymmetry is formed by maternal signals, the asymmetrically localized factors for establishing the AP axis in the embryos are zygotically induced. This is different from the long-germ insects like *Drosophila* and *Nasonia*, where maternal factors specify the AP polarity and expression of zygotic genes serves to maintain the AP axis. *Tribolium* thus provides a useful example for interpreting how the AP axis forms in other short/intermediate germ insects including the pea aphid.

### *Ap*-*hb*: a potential anterior determinant during viviparous development

In the asexual pea aphid, surprisingly, we find that the maternal *hunchback* (*Ap-hb*) mRNA is first localized to the anterior pole of stage-0 oocytes during early oogenesis (Huang et al. 2010). Anterior localization of *Ap-hb* can then be continuously detected in the developing oocytes and syncytial embryos prior to blastoderm formation (Figs. 4a; 5b) (Duncan et al. 2013; Huang et al. 2010). The localization pattern of *Ap-hb* very much resembles that of *bcd* in *Drosophila.* However, the pea aphid is missing a *bcd* orthologue (The International Aphid Genomics Consortium 2010). With the exception of *Ap-hb*, we have not found other reports of *bcd*-mimic localization of *hb* in either long- or short-germ insects (Kimelman and Martin 2012). For example, in the long-germ *Drosophila*, *Nasonia*, and *Apis mellifera* (honeybee), maternal *hb* mRNA is uniformly distributed in the oocytes and newly laid eggs (Fig. 5b) (Margolis et al. 1994; Pultz et al. 2005; Tautz et al. 1987; Wilson and Dearden 2011), and in the short-germ *Schistocerca* and *Oncopeltus*, anterior localization of the maternal *hb* is identified neither in the oocytes nor the uncellularized eggs (Liu and Kaufman 2004; Patel et al. 2001). Together with the finding of anterior localization of *Mp*-*hb*, an orthologue of *hb* in the green peach aphid, this strongly suggests that anterior localization of *hb* in the oocytes and early embryos is specific to the aphids (Chung et al. 2018; Huang et al. 2010). Whether this also applies to other non-aphid insects with parthenogenetic and viviparous reproduction requires further investigation.

WISH experiments using antisense riboprobes of *Ap-hb* in the ovaries of sexual pea aphids show that transcripts of *Ap-hb* are also restricted to the anterior pole of the developing oocytes. After fertilization, though, maternal *Ap-hb* becomes less concentrated in the egg anterior while zygotic *Ap-hb* is distributed along the embryonic primordium located in the egg posterior, the canonical location for formation of short-germ embryos (Figs. 4a; 5b) (Duncan et al. 2013). This suggests that (1) both asexual and sexual pea aphids utilize the anterior localization of *Ap-hb* to specify the anterior identity of oocytes and, perhaps, of newly laid eggs; that (2) viviparous embryos continue to employ the same mechanism to determine both the egg anterior and the embryonic anterior; but that (3) oviparous embryos, like other short-germ embryos formed in the egg posterior, do not make use of *Ap-hb* localization to the egg anterior (Liu and Kaufman 2005).

To date, although platforms of WISH and immunostaining have been established for detecting gene expression in the pea aphids (Chang et al. 2008; Chung et al. 2014; Lin and Chang 2016), tools for dissecting functions of genes in aphid embryos have not been successfully developed. It has therefore been difficult to uncover how *Ap-hb* operates for anterior determination. Nevertheless, expressions of *Ap-otd* and *Ap-cad* (Fig. 5c, d), both of which are conserved orthologues of the anterior gene *otd* and the posterior gene *cad* in insects (Lynch 2014), provide evidence to support the contribution of *Ap-hb* to anterior specification in viviparous embryos. First, the initial expression of *Ap-otd* is detected in the thickened germ band located near the egg anterior (Fig. 5c) (Huang et al. 2010), suggesting that egg anterior and embryonic anterior are formed toward the same direction. Second, *Ap-cad* expression detected at the opposite site of the *Ap-otd* expression in the germ band further verifies that the *Ap-otd*-positive region signifies the embryonic anterior (Fig. 5d) (Chang et al. 2013; Duncan et al. 2013). Additionally, the appearance of *Ap-otd* following *Ap-hb* localization implies that determination of the egg anterior positively regulates the formation of the embryonic anterior (Fig. 5b, c) (Huang et al. 2010). If this is the case, the process in viviparous development resembles the mode of anterior specification in long-germ insects such as *Drosophila* and *Nasonia* (Lynch et al. 2006; Riechmann and Ephrussi 2001).

### Vas and Nos: potential regulators of posterior development during viviparous development

With regard to posterior development, we have not found a gene whose products are localized to the posterior pole—the pole opposite to that of *Ap-hb* localization—in the nascent oocytes of the viviparous pea aphid. Signals detected by cross-reacting antibodies against Vas and Nos indicate that posterior localization of these two proteins first appears in eggs undergoing nuclear division, a sign of embryogenesis initiation (Miura et al. 2003). Prominent localization of Vas and Nos has not to date been detected in the oocyte posterior (Chang et al. 2006). Expression of *Ap-cad* is initially identified in the newly formed germ cells and blastodermal cells in the posterior region of the blastoderm (Chang et al. 2013). These results suggest that the AP axis of both oocytes and embryos is progressively established during viviparous development. However, we cannot exclude the possibility that there is a determinant being localized synchronously with *Ap-hb* in the oocyte posterior. If asymmetric localization of distinct molecules is still employed to establish the AP axis in the viviparous pea aphid, it will be interesting to explore whether Vas and Nos are the determinants for posterior specification or they are the downstream targets of a posterior determinant. Their expression patterns, however, do suggest that they are likely involved in the regulation of posterior development.

Until we can successfully employ tools like RNAi or CRISPR-Cas9 to dissect gene functions in the pea aphid embryos, we cannot conclude that Vas and Nos are determinants for posterior specification. At present, the developmental roles of *Ap-hb*, *Ap-otd*, *Ap-cad*, *Ap-vas*, and *Ap-nos* in pea aphids can only be inferred by expression data as well as conserved roles of their orthologues in *Drosophila* and other insects. Additional dissection of functions of these and other developmental genes will help to clarify (1) whether specification of the anterior identity of both eggs and embryos depends on *Ap-hb*; (2) whether *Ap-hb* is an upstream regulator of *Ap-otd*; (3) whether *Ap*-*vas* and *Ap-*Nos proteins specify the posterior axes of both eggs and embryos; and (4) whether *Ap-cad* is a downstream target of the products encoded by the *Ap-vas* and *Ap-nos* genes. Transcriptome analysis of the posterior genes in the pea aphid, including conserved and novel, should also provide more candidates for functional examination.

### Germline expression of *Ap*-*hb*/*Ap*-Hb in the viviparous pea aphid

Functional analysis of developmental genes in the pea aphid, if successfully developed, will provide answers to another question: how does *Ap-hb* regulate germline development? This question is prompted by the finding that *Ap-hb* mRNA and protein, in addition to their conserved expression in the neural system, are identified in the germ cells throughout embryogenesis in the viviparous pea aphid (Fig. 4) (Chung et al. 2018). Germline expression of *Mp-hb* can also be detected in viviparous green peach aphids, but a germline role for *hb* has not been reported outside Aphidoidea. Whether *Ap-hb* and *Mp-hb* are expressed in the germline of oviparous embryos remains unknown. The available evidence suggests that germline expression of *hb*, like anterior localization of *hb*, is a derived characteristic among the viviparous aphids. The absence of *Ap-hb* and *Ap*-Hb in the preformed germ plasm implies that the *Ap-hb* gene is not involved in germline specification (Chung et al. 2018; Huang et al. 2010). We note that germline expression of *Ap-hb/Ap*-Hb is detected soon after the blastoderm is formed, during which anterior localization of the maternal *Ap-hb* is missing (Figs. 4a, a'; 5b) (Chung et al. 2018). This suggests that germline expression of *Ap-hb* is zygotic. Additionally, if germline *Ap-hb* mRNA is synthesized for anterior localization of the oocytes in the embryonic gonads, germ cells—precursors of the oocytes—could already begin producing *Ap-hb* once they are settled within the germaria after katatrepsis. Early initiation of germline expression of *Ap-hb*, when germ cells are newly formed, also implies that *Ap-hb* may participate in the maintenance of germline development during embryogenesis.

## Conclusion and evolutionary aspects

In this review, we have discussed germline specification and axis determination in the pea aphids, focusing in particular on the asexual and viviparous morphs. Embryos of asexual pea aphids have an extending abdomen before katatrepsis and are therefore categorized as short-germ embryos, whose abdominal segments are gradually established during mid-embryogenesis (stages 7 to 14). Prior to abdominal extension (stages 3 to 6), however, the blastoderm occupies most of the egg. In this feature, aphid embryogenesis is more similar to that of long-germ embryos, whose blastoderm length is almost equivalent to the egg length. This “long germ-like” feature is not observed in other studied short-germ insects such as grasshoppers and crickets, whose embryonic primordia only occupy a small proportion of the egg posterior (Donoughe and Extavour 2016; Patel et al. 2001; Schröder et al. 2008).

In combining characteristics typical of development in both short- and long-germ embryos, viviparous development in the pea aphid exhibits three noteworthy features of early development. First, germline specification is driven by a preformed germ plasm expressing the conserved germline marker Vas (Chang et al. 2006). This is so far the sole example identified among hemimetabolans of Vas being employed as a component of the preformed germ plasm. Second, specification of the anterior identity of oocytes and embryos takes place in the same region and toward the same direction. The oocyte anterior is characterized by *Ap-hb* localization and the embryo anterior by *Ap-otd* expression (Huang et al. 2010). This differs from other studied short-germ insects, including the sexual pea aphids (Duncan et al. 2013; Lin et al. 2014), whose progeny develops oviparously and whose embryonic primordia arise from a small proportion in the egg posterior. Third, the mRNA of *hb* and its translated protein are expressed in the germ cells throughout embryogenesis (Chung et al. 2018). Expression of *hb* in the neural system during development is a highly conserved feature among insects, yet to date, germline expression of *hb* has only been identified in embryos of asexual aphids.

Identification of a Vas-positive germ plasm in oviparous oocytes of sexual pea aphid, such as the germline expression of *Ap-hb* mentioned above, is unexpected (Lin et al. 2014). These results suggest that sexual pea aphids, like other sexually reproduced hemimetabolans, might specify germ cells with the more ancestral approach, termed the signal-induction mode (Extavour and Akam 2003). After asexual vivipary evolved, the germ plasm–driven mode then co-evolved for development within the viviparous morphs and was eventually also utilized for development during oviparous reproduction. Afterwards, it was inherited by the oviparous morphs. However, we cannot exclude the possibility that germ plasm–driven development evolved simultaneously in viviparous and oviparous reproduction in aphids.

With regard to the anterior localization of *hb*, we hypothesize that it might be a feature that evolved in response to the invading endosymbiont *Buchnera*. Allowing precise invasion of endosymbionts in the embryo posterior from gastrulation onward requires setting the orientations of the eggs and embryos before the endosymbionts can invade: localization of *hb* transcripts may be utilized to label the egg and embryo anterior for this purpose. Likewise, posterior localization of Vas/Nos may fulfill the same purpose through a strategy of asymmetric localization.

In summary, we propose that the parthenogenic viviparity, an evolutionary novelty, drove the evolution of observed developmental novelties in germline specification and AP axis determination in the viviparous pea aphid. Whether establishment of the dorsal-ventral (DV) axis also exhibits asymmetric localization requires further investigation. To dissect developmental mechanisms in depth, the making of transgenic aphids and the development of effective approaches for dissecting gene functions are crucial. As more unique features of aphid development are uncovered, we believe they will serve the foundation for inventing more aphid-specific strategies to suppress the expansion of aphids.

## Data Availability

All data is provided in the text and the figures.
